# Relationship Between Vitamin D and Hormones Important for Human Fertility in Reproductive-Aged Women

**DOI:** 10.3389/fendo.2021.666687

**Published:** 2021-04-14

**Authors:** Chang Chu, Oleg Tsuprykov, Xin Chen, Saban Elitok, Bernhard K. Krämer, Berthold Hocher

**Affiliations:** ^1^ Fifth Department of Medicine (Nephrology/Endocrinology/Rheumatology), University Medical Centre Mannheim, University of Heidelberg, Heidelberg, Germany; ^2^ Department of Nephrology, Charité - Universitätsmedizin Berlin, Berlin, Germany; ^3^ Institut für Laboratoriumsmedizin Berlin IFLb, Berlin, Germany; ^4^ Department of Nephrology and Endocrinology/Diabetology, Klinikum Ernst von Bergmann, Potsdam, Germany; ^5^ Key Laboratory of Study and Discovery of Small Targeted Molecules of Hunan Province, School of Medicine, Hunan Normal University, Changsha, China; ^6^ Institute of Medical Diagnostics, IMD Berlin, Berlin, Germany; ^7^ Reproductive and Genetic Hospital of CITIC-Xiangya, Changsha, China

**Keywords:** total 25-hydroxyvitamin D, measured free 25-hydroxyvitamin D, fertility, hormones, reproductive-aged women

## Abstract

Vitamin D deficiency is very common in women of reproductive age. Studies in animals suggests a link between vitamin D and reproductive hormone biosynthesis. A systematic analysis of the correlation of reproductive hormones in reproductive-aged women with both total and free vitamin D was, however, not done so far. This cross-sectional study was performed in 351 healthy reproductive age Caucasian women (median age, 28.0 years; interquartile ranges, 24.7-31.0 years). We measured serum levels of both total and free 25(OH)D, endocrinological, hematological and biochemical parameters. Spearman’s rank correlations were performed to assess the correlation between 25(OH)D metabolites and selected parameters. Total vitamin D and free vitamin D measurements correlated well (rho=0.912, p < 0.0001). Both total 25(OH)D and free 25(OH)D showed significant negative correlation with FAI (rho=-0.229, p<0.0001 and rho=-0.195, p<0.0001 for total and free 25(OH)D, respectively); LH (rho=-0.177, p=0.001 and rho=-0.114, p=0.04 for total and free 25(OH)D, respectively), testosterone (rho=-0.174, p=0.001 and rho=-0.190, p<0.0001 for total and free 25(OH)D, respectively) and AMH (rho=-0.130, p=0.015 and rho=-0.107, p=0.047 for total and free 25(OH)D, respectively). Our study showed comparable correlations of both total and free 25(OH)D with endocrinological parameters, i.e. inverse correlations with free androgen index, luteinizing hormone, testosterone, LH/FSH ratio, androstenedione and anti-Müllerian hormone, and also with hematological and biochemical parameters, i.e. inverse correlations with erythrocytes, hsCRP and leukocytes count, and positive correlation with transferrin saturation, mean corpuscular hemoglobin and mean corpuscular volume in healthy reproductive age women.

## Introduction

Vitamin D deficiency is very common in women of reproductive age (1, 2). The physiological role of vitamin D in reproduction remains ambiguous. Early animal experiments found that 25(OH)D deficiency rats showed a compromised mating behavior, reduced fertility rates, decreased litter sizes and impaired neonatal growth, suggested the importance of vitamin D in reproduction (3–5). In human, low maternal vitamin D status has been associated with adverse maternal and fetal outcomes, including preeclampsia (6, 7), gestational diabetes mellitus (8, 9), small for gestational age (10) low birth weight (11).

Vitamin D appears to function through a single vitamin D receptor (VDR), which also has been identified in human female several reproductive tissues (12). Several studies reported that vitamin D deficiency affects both insulin secretion and insulin resistance. Insulin resistance is a hallmark of the polycystic ovarian syndrome, one of the most common endocrine disorders that affects reproductive age women (13–16).

As of today, measurements of total 25(OH)D are the most widely used way to assess vitamin D status in daily clinical practice. Serum 25(OH)D consists of three main metabolites, about 85 to 90% are bound to its specific carrier-vitamin D bound protein (DBP), about 10 to 15% are bound to serum albumin, only less than 0.1% exists as fully free unbound form (17, 18). However, only free vitamin D is bioactive, because it can pass freely the lipophilic cell membrane and interacts with the nuclear vitamin D receptor. Total vitamin D concentrations depends DBP concentrations, which depends itself on liver function, kidney function, endocrine status and even ethnicity rather than mineral-bone metabolism (19–22). Thus, total vitamin D concentrations may not entirely reflect the vitamin D status due to its above listed confounding factors. Free 25(OH)D might be a more reliable marker of vitamin D status in the body (23). This hypothesis is supported by recent studies (18, 24).

The associations of total 25(OH)D with sex steroid hormones were inconsistently reported in either women with PCOS or healthy women (25–28). We speculate that disparities in DBP levels in previous studies may led to the inconsistent associations between vitamin D and sex hormones. To the best of our knowledge, no information is available in human on the correlations of both total 25(OH)D and directly measured free 25(OH)D with reproduction related endocrinological parameters. We thus carried out a cross sectional study to compare total 25(OH)D and directly measured free 25(OH)D in a relatively large cohort of healthy reproductive age women (n=351). We evaluated correlations of 25(OH)D metabolites with reproduction related endocrinological parameters including follicle-stimulating hormone (FSH), luteinizing hormone (LH), thyroid hormones, anti-Müller hormone (AMH), sex steroid hormones and also hematological and biochemical parameters.

## Methods

### Study Participants

The study was a part of the Berlin Birth Cohort (BBC) study (29–31). This study was approved by the local ethical committee of the Charité - Universitätsmedizin Berlin, Germany. The study cohort consisted of 351 healthy Caucasian women of reproductive age asking for endocrine evaluation getting pregnant in four endocrine outpatients’ facilities in Berlin, Germany. Blood was taken between day 3 and 6 of the menstrual cycle. Patients with hypertension, any sign of heart/liver/kidney diseases, diabetes type 1 or 2 or any chronic disease such as rheumatoid arthritis and obesity were not asked to participate. All patients gave their written informed consent.

### Measurement of 25(OH)D Metabolites

The serum concentrations of total 25(OH)D (25-hydroxyvitamin D2 and D3) was measured by Abbott Architect i2000 (Abbott Laboratories, Wiesbaden, Germany) using Architect 25-OH Vitamin D chemiluminescent microparticle immunoassay (REF 5P02, Abbott Diagnostics, Wiesbaden, Germany), as previously described (32). Free 25(OH)D (25-hydroxyvitamin D2 and D3) was measured using a commercial kit from Future Diagnostics Solutions B.V. (Wijchen, Netherlands) distributed by DIASource Immunoassays, Louvain-la-Neuve, Belgium), according to the instructions of the manufacturer. The limit of blank (LoB) and the limit of detection (LoD) are determined based on CLSI EP17-A2, and intermediate precision and repeatability is determined based on CLSI EP05-A3 (https://www.diasource-diagnostics.com/var/ftp_diasource/IFO/KAPF1991.pdf).

### Measurement of Biochemical and Endocrine Parameters

Hematological and biochemical measurements including hemoglobin, erythrocytes, leukocytes, thrombocytes, mean corpuscular hemoglobin concentration (MCHC), mean corpuscular hemoglobin (MCH), mean corpuscular volume (MCV), hematocrit, red cell distribution width (RDW), high-sensitivity C-reactive protein (hsCRP), liver function, aspartate transaminase (AST), alanine transaminase (ALT), gamma-glutamyl transferase (GGT), thrombophilia parameters (APC-resistance, Antithrombin, Protein C, Protein S), anemia related parameters (Vitamin B12, Holotranscobalamin, Fe, Ferritin, Folic acid, Transferrin, Transferrin saturation).

Sex hormones and their metabolites including LH, FSH, estradiol (E2), estrone (E1), estrone sulfate (E1S), Progesterone (P4), 17-hydroxyprogesterone (17α-OHP), prolactin (PRL), testosterone (T), dihydrotestosterone (DHT), androstenedione (A4), dehydroepiandrosterone sulfate (DHEAS), AMH, androstenediol (Adiol), sex hormone-binding globulin (SHBG). The free androgen index (FAI) was calculated as (total testosterone × 100)/SHBG. Thyroid function parameters including free- triiodothyronine (fT3), thyroxin (T4), thyroid stimulating hormone (TSH) and thyroid peroxidase (TPO). Additional metabolites of steroid hormones - cortisol was also measured in this study.

Routine hematological, coagulation and clinical chemistry parameters were analyses on the following laboratory systems: Beckman Coulter UniCel^®^ DxH 800 analyzer (Beckman Coulter, Brea, CA, USA); Sysmex CS-5100 System (Siemens, Germany); Beckman Coulter UniCel^®^ DxC 800 Synchron Clinical Systems (Beckman Coulter, Brea, CA, USA); Abbott Architect i2000sr analyzer (Abbott Diagnostics, Wiesbaden, Germany) and Abbott Architect c4000 analyzer (Abbott Diagnostics, Wiesbaden, Germany) using to the manufacturers’ instructions. For the analyzed endocrine parameters details are provided in **Supplementary Table 1**.

All biochemical and endocrine parameters measurements were analyzed in a certified clinical laboratory, IFLb – Institute für Laboratoriumsmedizin Berlin (https://www.iflb.de/das-labor/), which has committed to quality management in accordance with the requirements of the DIN EN 15189 standard, verified by regular system and process reviews and monitoring by the German Accreditation Service (DAkkS) (accreditation number D-ML-13224-01). All measurements are subject to daily quality controls. External standards provided by an organization of the German clinical chemistry association are included in the daily measurements. All methods were performed in accordance with relevant guidelines and regulations.

### Statistical Analysis

Each parameter was examined for normality using the D’Agostino test, and most parameters were found to be not normally distributed (p <0.05). Descriptive statistics are presented as medians (interquartile ranges). Spearman’s rank correlation coefficients were performed to assess the correlation between 25(OH)D metabolites and selected hematological, biochemical and endocrinological parameters. Correlations were regarded significant if p-values were lower than 0.05. All analysis was performed using SPSS version 25.0 (Chicago, IL, USA) and GraphPad Prism version 8.0 (San Diego, CA, USA).

## Results

### Study Participant Characteristics

The study recorded 508 reproductive age women, 157 of them were excluded because of various health problems after clinical evaluation (45 with autoimmune thyroiditis; 30 anemia; 24 leukocytosis; 42 abnormal liver function; 11 thrombopenia; four subclinical hyperthyroidisms; one unknown). In 351 health women, the median age was 28.0 years (interquartile ranges: 24.7-31.0 years). 57.3 percent of participants (n=201) had 25(OH)D levels below 20 ng/mL. The median (interquartile ranges) of total 25 (OH)D and free 25 (OH)D were 18.37 ng/mL (12.65- 23.47) and 4.55 pg/mL (3.14-6.23), respectively. Baseline parameters are presented in **Table 1**, and baseline parameters of whole cohort (n=508) are presented in **Supplementary Table 2**.

**Table 1:** Main characteristics of the 351 healthy women (women without any abnormal laboratory parameter).

Parameter, units	N	Median (interquartile ranges)
Age, years	351	27.98 (24.72-31.00)
hsCRP, mg/L	337	0.98 (0.04-2.50)
**Hematological parameters**		
Hemoglobin, g/dl	351	13.20 (12.70-13.70)
Erythrocytes	351	4.50 (4.28-4.70)
Leukocytes	351	6.60 (5.60-7.70)
Thrombocytes	351	230.00 (204.00-266.00)
MCHC, g/dl	351	33.50 (33.10-34.00)
MCH, pg	351	29.60 (28.50-30.70)
MCV, fl	351	88.20 (85.60-91.10)
Hematocrit, %	351	39.30 (38.10-40.70)
RDW, %	351	13.20 (12.70-13.60)
**Liver function parameters**		
AST, U/l	333	19.40 (16.95-22.40)
ALT, U/l	333	14.60 (12.00-18.85)
GGT, U/l	343	14.40 (11.90-18.20)
**Thrombophilia parameters**		
APC- resistance	217	1.06 (1.02-1.11)
Antithrombin, %	217	105.30 (97.25-111.35)
Protein C, IU/dL	217	112.00 (100.00-125.00)
Protein S, %	217	87.00 (77.50-95.00)
**Thyroid function parameters**		
Free T3, pg/ml	346	2.84 (2.60-3.08)
T4, ng/dl	346	1.00 (0.92-1.08)
TSH, mU/L	346	1.24 (0.88-1.78)
Thyroid peroxidase, IU/ml	346	17.00 (11.00-24.00)
**Sex hormones related parameters**		
LH, mIE/ml	326	4.90 (2.98-7.10)
FSH, mIE/ml	324	4.35 (3.30-5.60)
Estradiol, pg/ml	350	40.00 (21.00-64.25)
Estrone, pg/ml	24	77.00 (53.75-102.25)
Estrone sulfate, ng/ml	22	2.65 (2.13-4.00)
Progesterone, ng/ml	350	0.20 (0.10-0.20)
17-hydroxyprogesterone, ng/ml	340	0.50 (0.40-0.70)
PRL, ng/ml	349	11.20 (8.10-15.95)
Testosterone, ng/ml	351	0.31 (0.25-0.41)
Dihydrotestosterone, pg/ml	350	240.50 (171.00-334.00)
Androstenedione, ng/ml	350	1.70 (1.30-2.20)
DHEA-S, ng/ml	351	2815.00 (2089.00-3864.00)
SHBG, nmol/l	350	60.45 (40.35-95.88)
FAI	350	1.70 (0.90-2.90)
Cortisol, µg/dl	349	8.00 (6.00-11.00)
Adiol, ng/ml	348	3.30 (2.00-4.98)
AMH, ng/ml	347	5.66 (2.99-9.22)
**Anemia related parameters**		
Vitamin B12, pg/ml	351	350 (273-447)
Holotranscobalamin, pmol/l	113	53.60 (43.35-74.35)
Fe, µg/dl	131	71.39 (50.80-98.36)
Ferritin, ng/mL	351	37.50 (24.40-55.70)
Folic acid, ng/ml	351	6.00 (4.20-9.10)
Transferrin, mg/dl	127	289.00 (264.00-320)
Transferrin saturation, %	127	18.00 (12.00-25.00)
**Vitamin D status**		
Total 25(OH)D, ng/ml	349	18.37 (12.65-23.47)
Free 25(OH)D, pg/ml	349	4.55 (3.14-6.23)

hsCRP, high-sensitivity C-reactive protein; MCHC, mean corpuscular/cellular hemoglobin concentration; MCH, mean corpuscular hemoglobin; MCV, mean corpuscular volume; RDW, red blood cell distribution width; AST, aspartate transaminase; ALT, alanine transaminase; GGT, Gamma-glutamyl transferase; APC-resistance, resistance to activated protein C; TSH, Thyroid-stimulating hormone; LH, Luteinizing hormone; FSH, Follicle-stimulating hormone; PRL, Prolactin; DHEA-S, Dehydroepiandrosterone sulfate; SHBG, Sex hormone-binding globulin; FAI, Free androgen index; AMH, Anti-Müllerian hormone.

### Associations Between Free 25(OH)D and Total 25(OH)D

**Figure 1:**
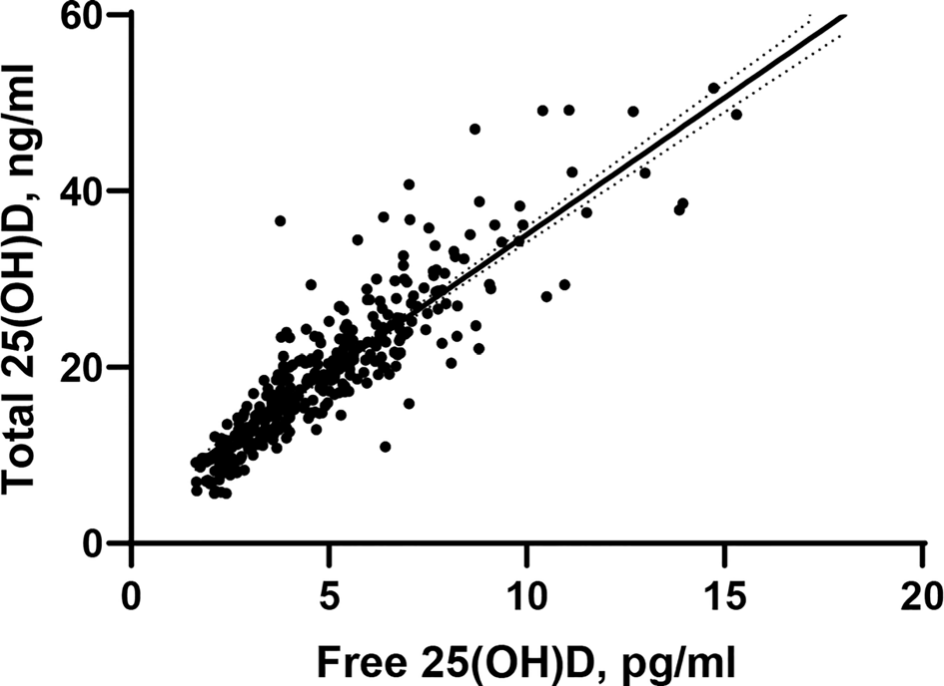
Scatter plots depicting bivariate associations between free 25(OH) D and total 25(OH)D. rho=0.912, p<0.0001.

The positive and strong correlation between total 25(OH)D and free 25(OH)D was statistically significant (rho=0.912, p < 0.0001, **Figure 1**).

### Correlations Between Total 25(OH)D and Free 25(OH)D With Endocrinological Parameters

Spearman’s correlations between endocrinological parameters and 25(OH)D metabolites are presented in **Table 2**. Both total 25(OH)D and free 25(OH)D showed weak but still significant negative correlations with FAI (rho=-0.229, p<0.0001 and rho=-0.195, p<0.0001 for total and free 25(OH)D, respectively); LH (rho=-0.177, p=0.001 and rho=-0.114, p=0.04 for total and free 25(OH)D, respectively), testosterone (rho=-0.174, p=0.001 and rho=-0.190, p<0.0001 for total and free 25(OH)D, respectively), androstendion (rho=-0.132, p=0.014 and rho=-0.121, p=0.024 for total and free 25(OH)D, respectively) and AMH (rho=-0.130, p=0.015 and rho=-0.107, p=0.047 for total and free 25(OH)D, respectively). Total 25(OH)D showed a significant negative association with DHEAS (rho=-0.109, p=0.041), however, free 25(OH)D showed no significant correlation with DHEAS (rho=-0.085, p=0.111). Free 25(OH)D showed weak correlation with PRL (rho=-0.119, p=0.027), whereas total 25(OH)D showed no such significant correlation. Besides, total 25(OH)D levels showed a stronger positive correlation with SHBG (rho=0.195, p<0.001) compared to the free 25(OH)D levels (rho=0.156, p=0.004).

**Table 2:** Bivariate correlations between total and free 25(OH)D and selected hormones sorted in ascending order according to spearman’s rank correlation coefficient (rho) for total 25(OH)D in 351 healthy women (women without any abnormal laboratory parameter).

Parameter, units	Total 25(OH)D, ng/ml	Free 25(OH)D, pg/ml	Spearman's rho color gradient
FAI	-0.229^**^	-0.195^**^	-0.15>rho>-0.25, p<0.05
LH, mIE/ml	-0.177^**^	-0.114^*^	-0.10>rho≥-0.15, p<0.05
Testosterone, ng/ml	-0.174^**^	-0.190^**^	-0.05>rho≥-0.10, p<0.05
LH/FSH ratio	-0.139^*^	-0.116^*^	0.00>rho≥-0.05, p<0.05
Androstenedione, ng/ml	-0.132^*^	-0.121^*^	0.00≤rho<0.05 or p>0.05
AMH, ng/ml	-0.130^*^	-0.107^*^	0.05≤rho<0.10, p<0.05
DHEAS, ng/ml	-0.109^*^	-0.085	0.10≤rho<0.20, p<0.05
PRL, ng/ml	-0.099	-.119^*^	0.20≤rho<0.30, p<0.05
TSH, mU/L	-0.089	-0.088	0.30≤rho<0.40, p<0.05
17-Hydroxyprogesterone, ng/ml	-0.089	-0.093	
T4, ng/dl	-0.086	-0.047	
FSH, mIE/ml	-0.075	0.006	
Progesterone, ng/ml	-0.070	-0.069	
DHT, pg/ml	-0.068	-0.048	
Adiol, ng/ml	-0.048	-0.048	
Free T3, pg/ml	-0.024	-0.083	
Estradiol, pg/ml	-0.021	0.021	
Estrone, pg/mL	0.037	0.167	
Cortisol, µg/dl	0.047	0.020	
SHBG, nmol/l	0.195^**^	0.156^**^	
E1S, ng/mL	0.302	0.404	

**Correlation is significant at the 0.01 level; *correlation is significant at the 0.05 level. FAI, Free androgen index; LH, Luteinizing hormone; AMH, Anti-Müllerian hormone; TSH, Thyroid-stimulating hormone; DHEA-S, Dehydroepiandrosterone sulfate; PRL, Prolactin; FSH, Follicle-stimulating hormone; DHT, Dihydrotestosterone; SHBG, Sex hormone-binding globulin; E1S, Estrone sulfate.

### Correlations Between Total 25(OH)D and Free 25(OH)D With Hematological and Biochemical Parameters

Regarding the associations with hematological parameters, both 25(OH)D metabolites showed weak associations with erythrocytes, leukocytes (rho=-0.228, p<0.0001 and rho=-0.154, p=0.004 for total 25(OH)D with erythrocytes and leukocytes, respectively, compared to rho=-0.227, p<0.0001 and rho=-0.168, p=0.002 for free 25(OH)D with erythrocytes and leukocytes, respectively). Both total 25(OH)D and free 25(OH)D showed significant negative correlations with hsCRP (rho=-0.167, p=0.002 and rho=-0.219, p<0.0001 for total and free 25(OH)D, respectively). Although both 25(OH)D metabolites showed positive correlation MCH and MCV, free 25(OH)D showed better correlations (rho=0.260, p< 0.0001 and rho=0.312, p< 0.0001 with MCH and MCV, respectively, compared to rho=0.232, p< 0.0001 and rho=0.267, p< 0.0001 for total 25(OH)D with MCH and MCV, respectively). Whereas, free 25(OH)D but not total 25(OH)D showed negative correlation with thrombocytes (rho=-0.134, p=0.012) and protein C (rho=-0.148, p=0.030). Both free 25(OH)D and total 25(OH)D showed significant correlations with folic acid than total 25(OH)D (rho= 0.250, p<0.0001 and rho=0.220, p<0.0001 with free and total 25(OH)D, respectively), and transferrin saturation (rho=0.193, p=0.031 and rho= 0.223, p=0.012 with free and total 25(OH)D, respectively). Free 25(OH)D but not total 25(OH)D showed significant positive correlation with Vitamin B12 (rho=0.150, p=0.005 and rho= 0.076, p= 0.159 with free and total 25(OH)D, respectively).

All these correlations remained basically similar also after inclusion of 157 women having not completely normal clinical laboratory parameters (45 with autoimmune thyroiditis; 30 with anemia; 24 with leukocytosis; 42 with unnormal liver function; 11 with thrombopenia; four women with subclinical hyperthyroidism, one with unknown reason). The results of bivariate correlations of the 508 women are given in **Supplementary Tables 3** and **4**.

## Discussion

This cross-sectional study compared total 25(OH)D and directly measured free 25(OH)D in healthy reproductive age women. Total vitamin D and free vitamin D measurements correlated well. Both showed comparable correlations with reproduction related endocrinological, hematological parameters, liver function parameters, thrombophilia parameters and anemia related parameters (see **Tables 2** and **3**).

**Table 3:** Bivariate correlations between total and free 25(OH)D and hematological and biochemical parameters sorted in ascending order according to according to spearman’s rank correlation coefficient (rho) for total 25(OH)D in 351 healthy women (women without any abnormal laboratory parameter).

Parameter, units	Total 25(OH)D, ng/ml	Free 25(OH)D, pg/ml	Spearman's rho color gradient
Erythrocytes	-0.228^**^	-0.227^**^	-0.15>rho>-0.25, p<0.05
hsCRP, mg/L	-0.167^**^	-0.219^**^	-0.10>rho≥-0.15, p<0.05
Leukocytes	-0.154^**^	-0.168^**^	-0.05>rho≥-0.10, p<0.05
Protein S	-0.142^*^	-0.087	0.00>rho≥-0.05, p<0.05
RDW	-0.136^*^	-0.125^*^	0.00≤rho<0.05 or p>0.05
GGT, U/l	-0.109^*^	-0.076	0.05≤rho<0.10, p<0.05
Transferrin, mg/dl	-0.137	-0.132	0.10≤rho<0.20, p<0.05
Protein C	-0.106	-0.148^*^	0.20≤rho<0.30, p<0.05
ALT, U/l	-0.079	-0.063	0.30≤rho<0.40, p<0.05
Antithrombin	-0.057	-0.009	
Thrombocytes	-0.052	-0.134^*^	
Holotranscobalamin, pmol/l	-0.014	0.007	
MCHC, g/dl	-0.012	-0.012	
Hematokrit	-0.003	0.040	
Thyreoidale Peroxidase	0.002	0.000	
Hemoglobin, g/dl	0.002	0.041	
AST, U/l	0.014	0.074	
APC- resistance	0.037	0.092	
Vitamin B12, pg/ml	0.076	0.150^**^	
Ferritin, ng/mL	0.099	0.112^*^	
Age, years	0.114^*^	0.106^*^	
Fe, µg/dl	0.190^*^	0.167	
Folic acid, ng/ml	0.220^**^	0.250^**^	
Transferrin saturation, %	0.223^*^	0.193^*^	
MCH, pg	0.232^**^	0.260^**^	
MCV, fl	0.267^**^	0.312^**^	

**Correlation is significant at the 0.01 level; *correlation is significant at the 0.05 level. RDW, red blood cell distribution width; hsCRP, high-sensitivity C-reactive protein; ALT, alanine transaminase; GGT, Gamma-glutamyl transferase; AST, aspartate transaminase; MCHC, mean corpuscular/cellular hemoglobin concentration; APC-resistance, resistance to activated protein C; MCH, mean corpuscular hemoglobin; MCV, mean corpuscular volume.

Increasing evidences show that vitamin D may play an important role in regulating female fertility (33–36). Low vitamin D status is associated with adverse maternal and fetal outcomes (6–11), and is involved in the development of specific gynecological conditions that affecting fertility, such as endometriosis and polycystic ovarian syndrome (PCOS) (14, 37, 38). Several animal and human studies have linked vitamin D metabolism with sex steroid synthesis (25, 39). In the present study we observed that both total and free 25(OH)D were inversely correlated with FAI, LH, total testosterone, AMH, androstenedione, TSH, and positively correlated with SHBG in reproductive-age women.

It is a key finding of the current study that laboratory parameters describing androgens such as free androgen index (FAI), total testosterone and androstenedione are among the parameters with the strongest negative correlation between either free or total vitamin D in reproductive-age Caucasian women. Testosterone has been extensively studied in male, and considerable evidence supports the influence of vitamin D on semen quality through the regulation of calcium metabolism and testosterone production (39). However, few studies have focused on the effects of vitamin D on testosterone in women. Our results showed that both total and free 25(OH)D were negatively correlated with testosterone. Our data are in agreement with another study showing a negative correlation between total 25 (OH)D and free T as well as total T in the follicular fluid of healthy women (40) and with a meta-analysis of studies in women with PCOS (41) showing likewise an inverse relationship of testosterone to total vitamin D. There is, however, a small study in healthy women showing a positive correlation between androgens and total vitamin D (25). Given the size of this study – eight times smaller than our study and the other studies in PCOS women and the study analyzing ovarian fluid, see above, it is more likely that this small study was simply underpowered.

From a biochemical aspect, both androgens and vitamin D belong to the steroid family. As mentioned above, we report a negative correlation between laboratory parameters describing androgens and vitamin D. A potential explanation of such a correlation could be derived from the ability of vitamin D to act as a regulator of a number of enzymes involved in the regulation of the production of adrenal steroid hormones including adrenal androgens as well as ovarian sex hormones (42). Sex hormones are produced in the gonads either by *in situ* synthesis from cholesterol or by enzyme catalyzed conversion of androstenedione or DHEA which are excreted to the circulation from the adrenal gland. Androgens (e.g. testosterone) are produced *via* reactions catalyzed by 17β-hydroxysteroid dehydrogenase (17β-HSD). The expression of this enzyme was reported to be regulated by 1α,25-dihydroxyvitamin D_3_ in human prostate cell lines and keratinocytes (43, 44). Moreover, 1α,25-dihydroxyvitamin D_3_ was reported to exert tissue-specific effects on androgen metabolism where it led to increased androgen production in breast cancer cells, however, dihydrotestosterone production was decreased in adrenocortical cells treated with 1α,25-dihydroxyvitamin D_3_ (45).

Our observational clinical data might suggest that treatment with vitamin D might be a way to improve the endocrine status in women with hyperandrogenism. However, two meta-analyses have reported controversial findings with respect to the effect of vitamin D supplementation on androgens in women with PCOS (46, 47). This could be due to two circumstances. First, the vitamin D status in these studies was determined using total vitamin D. Second, the optimal doses to improve the androgen status in humans are still unknown. Proper dose finding studies would be needed. However, this was not done. Vitamin D concentrations for an optimal androgen status, fertility rate and embryo development in humans are currently unknown. PCOS - beside high androgens - is also characterized by elevated LH and AMH (48, 49). Both are also inversely correlated to free and total vitamin D. This again underlines the possible importance of vitamin D to optimize the endocrine situation in women with PCOS. As mentioned above, this would require adequately designed dose-finding studies.

The present study also indicated an association between 25(OH)D and anemia related parameters, which is in line with some previous studies (50–54). And we also found that both vitamin D metabolites are negatively correlated with hsCRP, which draw attention to the hypothesis that low 25(OH)D maybe involved in the chronic inflammation. Low grade inflammation is an unfavorable factor when aiming to become pregnant and also harmful for the developing fetus (55–57).

The strength of correlation of free and total 25(OH)D and endocrinological, hematological and biochemical parameters was similar in healthy reproductive age women. Thus, both ways of analyzing the vitamin D status in healthy reproductive age women with regard to the evaluation of endocrine fertility parameters are suitable tools. The effect of estrogens on vitamin D binding protein and hence the relationship of free and total 25(OH)D is obviously negligible in a study population of healthy women with the same genetic background. This might be different in populations with mixed ethnic background and underlying diseases with known effects on serum vitamin D binding protein concentrations such as kidney diseases or liver diseases (5, 17). Kidney diseases and major liver diseases are usually rare in young women, thus the use of both ways of analysing the vitamin D status in this particular population is justified. If these young women get pregnant, the situation changes, then, the rising levels of female steroids hormones do substantially induce hepatic synthesis of vitamin D binding protein (58) and hence free 25(OH)D is much better correlated with markers of bone metabolism (calcium, BSAP), lipid metabolism (adiponectin, LDL cholesterol, LDL/HDL ratio) and kidney function (urea) than total 25(OH)D (32).

A strength of our study represents its large sample size of Caucasian women. Furthermore, we measured both total and free 25(OH)D. We acknowledge that a cross-sectional study design limits drawing conclusion on causality, and the lack of clinical data such as previous obstetric/gynecological history, body mass index and blood pressure represents a study limitation.

In conclusion, this is the first study to compare total 25(OH)D and directly measured free 25(OH)D in a relatively large cohort of healthy reproductive age women. Total and free vitamin D showed similar correlations with reproduction related endocrinological parameters, hematological parameters, liver function parameters, thrombophilia parameters and anemia related parameters in healthy reproductive age women. In particular androgens but also LH and AMH are inversely correlated with both free and total 25(OH)D.

In conclusion, this is the first study to compare total 25(OH)D and directly measured free 25(OH)D in a relatively large cohort of healthy reproductive age women. Total and free vitamin D showed similar correlations with endocrinological parameters, i.e. inverse correlations with free androgen index, luteinizing hormone, testosterone, LH/FSH ratio, androstenedione and anti-Müllerian hormone, and also with hematological and biochemical parameters, i.e. inverse correlations with erythrocytes, hsCRP and leukocytes count, and positive correlation with transferrin saturation, mean corpuscular hemoglobin and mean corpuscular volume in healthy reproductive age women.

## Data Availability Statement

The raw data supporting the conclusions of this article will be made available by the authors, without undue reservation.

## Ethics Statement

Written informed consent was obtained from the individual(s) for the publication of any potentially identifiable images or data included in this article.

## Author Contributions 

BH designed the study. CC, OT, XC, and SE collected the data. BH and CC did statistics. CC, BH, and BK wrote the manuscript. All authors contributed to the article and approved the submitted version.

## Conflict of Interest

The authors declare that the research was conducted in the absence of any commercial or financial relationships that could be construed as a potential conflict of interest.
