# Supplementary material for: Relationship Between Vitamin D and Hormones Important for Human Fertility in Reproductive-Aged Women
Source: Front Endocrinol (Lausanne). 2021 Apr 14;12:666687. doi: 10.3389/fendo.2021.666687 (PMC8081388; doi:10.3389/fendo.2021.666687)
Supplement: Supplementary file 1 [file Table_1.docx]

**Relationship between Vitamin D and Hormones important for human Fertility in reproductive-aged Women**

Chang Chu^1,2^, Oleg Tsuprykov^1,3^, Xin Chen^1,2^, Saban Elitok^1,4^, Bernhard K. Krämer^1^, Berthold Hocher^1,5,6,7^

^1^ Fifth Department of Medicine (Nephrology/Endocrinology/Rheumatology), University Medical Centre Mannheim, University of Heidelberg, Heidelberg, Germany.

^2^ Department of Nephrology, Charité - Universitätsmedizin Berlin, Campus Mitte, Berlin, Germany.

^3^ Institut für Laboratoriumsmedizin Berlin IFLb, Berlin, Germany.

^4^ Department of Nephrology and Endocrinology/Diabetology, Klinikum Ernst von Bergmann, Potsdam, Germany.

^5^ Key Laboratory of Study and Discovery of Small Targeted Molecules of Hunan Province, School of Medicine, Hunan Normal University, Changsha, China.

^6^ Institute of Medical Diagnostics, IMD Berlin, Berlin, Germany.

^7^ Reproductive and Genetic Hospital of CITIC-Xiangya, Changsha, China.

**Supplementary table 1.** Main characteristics of the whole cohort.

| **Parameter, units** | **N** | **Median (interquartile ranges)** |
| --- | --- | --- |
| Age, years | 508 | 27.82 (24.56-31.17) |
| hsCRP, mg/L | 491 | 1.10 (0.04-2.90) |
| **Hematological parameters** |  |  |
| Hemoglobin, g/dl | 507 | 13.10 (12.60-13.70) |
| Erythrocytes | 507 | 4.50 (4.30-4.74) |
| Leukocytes | 507 | 6.70 (5.60-8.20) |
| Thrombocytes | 507 | 231.00 (203.00-269.00) |
| MCHC, g/dl | 507 | 33.40 (33.00-33.90) |
| MCH, pg | 507 | 29.40 (28.20-30.40) |
| MCV, fl | 507 | 87.50 (84.60-90.50) |
| Hematocrit, % | 507 | 39.20 (37.80-40.70) |
| RDW, % | 507 | 13.30 (12.80-13.90) |
| **Liver function parameters** |  |  |
| AST, U/l | 487 | 20.10 (17.10-24.40) |
| ALT, U/l | 487 | 15.50 (12.10-21.30) |
| GGT, U/l | 498 | 14.80 (12.00-19.70) |
| **Thrombophilia parameters** |  |  |
| APC- resistance | 320 | 1.06 (1.01-1.10) |
| Antithrombin, % | 320 | 105.40 (98.23-112.38) |
| Protein C, IU/dL | 320 | 110.00 (99.25-125.00) |
| Protein S, % | 320 | 86.00 (78.00-95.00) |
| **Thyroid function parameters** |  |  |
| Free T3, pg/ml | 502 | 2.86 (2.60-3.10) |
| T4, ng/dl | 503 | 1.00 (0.92-1.09) |
| TSH, mU/L | 503 | 1.26 (0.89-1.85) |
| Thyroid peroxidase, IU/ml | 502 | 18.00 (12.00-28.25) |
| **Sex hormones related parameters** |  |  |
| LH, mIE/ml | 481 | 4.80 (2.95-6.80) |
| FSH, mIE/ml | 478 | 4.20 (3.30-5.30) |
| Estradiol, pg/ml | 507 | 40.00 (20.00-66.00) |
| Estrone, pg/ml | 27 | 85.00 (59.00-115.00) |
| Estrone sulfate, ng/ml | 24 | 2.70 (2.23-3.75) |
| Progesterone, ng/ml | 507 | 0.20 (0.10-0.20) |
| 17-hydroxyprogesterone, ng/ml | 492 | 0.50 (0.40-0.70) |
| PRL, ng/ml | 505 | 11.20 (7.65-15.75) |
| Testosterone, ng/ml | 508 | 0.31 (0.24-0.41) |
| Dihydrotestosterone, pg/ml | 506 | 237.00 (169.00-337.00) |
| Androstenedione, ng/ml | 505 | 1.70 (1.20-2.20) |
| DHEA-S, ng/ml | 506 | 2778.50 (2038.00-3806.75) |
| SHBG, nmol/l | 507 | 61.20 (38.20-97.90) |
| FAI | 507 | 1.70 (0.90-3.00) |
| Cortisol, µg/dl | 506 | 8.00 (6.00-11.00) |
| Adiol, ng/ml | 504 | 3.30 (2.10-4.90) |
| AMH, ng/ml | 501 | 5.53 (3.02-8.88) |
| **Anemia related parameters** |  |  |
| Vitamin B12, pg/ml | 508 | 349.00 (272.25-452.50) |
| Holotranscobalamin, pmol/l | 165 | 52.90 (42.40-75.35) |
| Fe, µg/dl | 188 | 67.78 (46.16-93.33) |
| Ferritin, ng/mL | 508 | 37.60 (22.98-57.78) |
| Folic acid, ng/ml | 507 | 5.90 (4.10-8.70) |
| Transferrin, mg/dl | 184 | 296.50 (267.25-323.75) |
| Transferrin saturation, % | 184 | 17.00 (10.00-23.00) |
| **Vitamin D status** |  |  |
| Total 25(OH)D, ng/ml | 506 | 16.78 (11.99-22.74) |
| Free 25(OH)D, pg/ml | 506 | 4.08 (2.93-5.67) |

Abbreviations: hsCRP, high-sensitivity C-reactive protein; MCHC, mean corpuscular/cellular hemoglobin concentration; MCH, mean corpuscular hemoglobin; MCV, mean corpuscular volume; RDW, red blood cell distribution width; AST, aspartate transaminase; ALT, alanine transaminase; GGT, Gamma-glutamyl transferase; APC-resistance, resistance to activated protein C; TSH, Thyroid-stimulating hormone; LH, Luteinizing hormone; FSH, Follicle-stimulating hormone; PRL, Prolactin; DHEA-S, Dehydroepiandrosterone sulfate; SHBG, Sex hormone-binding globulin; FAI, Free androgen index; AMH, Anti-Müllerian hormone;

**Supplementary table 2.** Bivariate correlations between total and free 25(OH)D and selected hormones sorted in ascending order according to rho value for total 25(OH)D in whole cohort (n=508).

| **Parameter, units** | **Total 25(OH)D, ng/ml** | **Free 25(OH)D, pg/ml** | **Spearman's rho color gradient** |
| --- | --- | --- | --- |
| FAI | -0.199** | -0.166** | -0.15>rho>-0.25, p<0.05 |
| LH, mIE/ml | -0.153** | -0.101* | -0.10>rho≥-0.15, p<0.05 |
| Testosterone, ng/ml | -0.131** | -0.143** | -0.05>rho≥-0.10, p<0.05 |
| AMH, ng/ml | -0.128** | -0.088 | 0.00>rho≥-0.05, p<0.05 |
| LH/FSH ratio | -0.119** | -0.100* | 0.00≤rho<0.05 or p>0.05 |
| Androstenedione, ng/ml | -0.104* | -0.083 | 0.05≤rho<0.10, p<0.05 |
| TSH | -0.100* | -0.121** | 0.10≤rho<0.20, p<0.05 |
| DHEAS, ng/ml | -0.100* | -0.078 | 0.20≤rho<0.30, p<0.05 |
| PRL, ng/ml | -0.074 | -0.093* | 0.30≤rho<0.40, p<0.05 |
| 17-Hydroxyprogesterone, ng/ml | -0.073 | -0.072 |  |
| T4 | -0.068 | -0.037 |  |
| FSH, mIE/ml | -0.063 | 0.012 |  |
| Adiol, ng/ml | -0.048 | -0.042 |  |
| DHT, pg/ml | -0.034 | -0.028 |  |
| Progesterone, ng/ml | -0.028 | -0.015 |  |
| Free T3, pg/ml | -0.027 | -0.068 |  |
| Estrone, pg/mL | -0.008 | 0.076 |  |
| Estradiol, pg/ml | 0.035 | 0.051 |  |
| Cortisol, µg/dl | 0.076 | 0.064 |  |
| SHBG, nmol/l | 0.182** | 0.139** |  |
| E1S, ng/mL | 0.283 | 0.360 |  |

**Correlation is significant at the 0.01 level; *Correlation is significant at the 0.05 level. Abbreviations: FAI, Free androgen index; LH, Luteinizing hormone; AMH, Anti-Müllerian hormone; TSH, Thyroid-stimulating hormone; DHEA-S, Dehydroepiandrosterone sulfate; PRL, Prolactin; FSH, Follicle-stimulating hormone; DHT, Dihydrotestosterone; SHBG, Sex hormone-binding globulin; E1S, Estrone sulfate.

**Supplementary table 3.** Bivariate correlations between total and free 25(OH)D and hematological and biochemical parameters sorted in ascending order according to rho value for total 25(OH)D in whole cohort (n=508).

|  | **Total 25(OH)D, ng/ml** | **Free 25(OH)D, pg/ml** | **Spearman's rho color gradient** |
| --- | --- | --- | --- |
| Erythrocytes | -0.191** | -0.176** | -0.15>rho>-0.25, p<0.05 |
| RDW | -0.182** | -0.176** | -0.10>rho≥-0.15, p<0.05 |
| Transferrin, mg/dl | -0.173* | -0.185* | -0.05>rho≥-0.10, p<0.05 |
| Leukocytes | -0.172** | -0.213** | 0.00>rho≥-0.05, p<0.05 |
| hsCRP, mg/L | -0.160** | -0.223** | 0.00≤rho<0.05 or p>0.05 |
| ALT, U/l | -0.117** | -0.095* | 0.05≤rho<0.10, p<0.05 |
| GGT, U/l | -0.115* | -0.101* | 0.10≤rho<0.20, p<0.05 |
| Protein C | -0.097 | -0.126* | 0.20≤rho<0.30, p<0.05 |
| Antithrombin | -0.070 | -0.049 | 0.30≤rho<0.40, p<0.05 |
| Thrombocytes | -0.066 | -0.113* |  |
| Protein S | -0.060 | -0.028 |  |
| Thyreoidale Peroxidase | -0.049 | -0.050 |  |
| AST, U/l | -0.046 | 0.015 |  |
| Holotranscobalamin, pmol/l | -0.029 | -0.014 |  |
| Hematokrit | 0.053 | 0.086 |  |
| MCHC, g/dl | 0.054 | 0.059 |  |
| Hemoglobin, g/dl | 0.062 | 0.098* |  |
| APC- resistance | 0.074 | 0.111* |  |
| Vitamin B12, pg/ml | 0.088* | 0.147** |  |
| Age, years | 0.090* | 0.077 |  |
| Ferritin, ng/mL | 0.129** | 0.129** |  |
| Fe, µg/dl | 0.220** | 0.236** |  |
| Folic acid, ng/ml | 0.245** | 0.258** |  |
| Transferrin saturation, % | 0.258** | 0.269** |  |
| MCH, pg | 0.261** | 0.273** |  |
| MCV, fl | 0.283** | 0.303** |  |

**Correlation is significant at the 0.01 level; *Correlation is significant at the 0.05 level. Abbreviations: RDW, red blood cell distribution width; hsCRP, high-sensitivity C-reactive protein; ALT, alanine transaminase; GGT, Gamma-glutamyl transferase; AST, aspartate transaminase; MCHC, mean corpuscular/cellular hemoglobin concentration; APC-resistance, resistance to activated protein C; MCH, mean corpuscular hemoglobin; MCV, mean corpuscular volume.

**Supplementary Table 4.** Methods and materials used for the measurement of endocrinological parameters.

| **Parameter, units** | **Method** | **Material / Notes** |
| --- | --- | --- |
| FAI | - | Calculation from SHBG and testosterone |
| LH, mIE/ml | CMIA | Serum |
| Testosterone, ng/ml | CMIA | Serum |
| LH/FSH ratio | - | Calculation from LH and FSH |
| Androstendion, ng/ml | EIA | Serum |
| AMH, ng/ml | EIA | Serum |
| DHEAS, ng/ml | CMIA | Serum |
| PRL, ng/ml | CMIA | Serum |
| TSH, mU/L | CMIA | Serum |
| 17-Hydroxyprogesterone, ng/ml | ELISA | Serum |
| T4, ng/dl | CMIA | Serum |
| FSH, mIE/ml | CMIA | Serum |
| Progesterone, ng/ml | CMIA | Serum |
| DHT, pg/ml | ELISA | Serum |
| Adiol, ng/ml | EIA | Serum |
| Free T3, pg/ml | CMIA | Serum |
| Estradiol, pg/ml | CMIA | Serum |
| Estrone, pg/mL | RIA | Serum |
| Cortisol, µg/dl | LIA | Serum |
| SHBG, nmol/l | CMIA | Serum |
| E1S, ng/mL | RIA | Serum |

All biochemical and endocrine parameters measurements were analyses in a certified clinical laboratory, IFLb - Laboratoriumsmedizin Berlin (https://www.iflb.de/das-labor/), which has committed to quality management in accordance with the requirements of the DIN EN 15189 standard, verified by regular system and process reviews and monitoring by the German Accreditation Service (DAkkS). Meanwhile, an accreditation with the German Accreditation Body under the accreditation number D-ML-13224-01 as a confirmation of the quality. All measurements are subject to daily quality controls. External standards provided by an organisation of the German clinical chemistry association are included in the daily measurements. Abbreviations: FAI, Free androgen index; LH, Luteinizing hormone; AMH, Anti-Müllerian hormone; TSH, Thyroid-stimulating hormone; DHEA-S, Dehydroepiandrosterone sulfate; PRL, Prolactin; FSH, Follicle-stimulating hormone; DHT, Dihydrotestosterone; SHBG, Sex hormone-binding globulin; E1S, Estrone sulfate. CMIA, Chemiluminescent microparticle immunoassay; EIA, Enzyme immunoassay; ELISA, enzyme-linked immunosorbent assay; RIA, Radio immunoassay; LIA, Chemiluminescence immunoassay.
